# Metagenomic analysis reveals the relationship between intestinal protozoan parasites and the intestinal microecological balance in calves

**DOI:** 10.1186/s13071-023-05877-z

**Published:** 2023-07-31

**Authors:** Yin Fu, Kaihui Zhang, Mengyao Yang, Xiaoying Li, Yuancai Chen, Junqiang Li, Huiyan Xu, Pitambar Dhakal, Longxian Zhang

**Affiliations:** 1 College of Veterinary Medicine, Henan Agricultural University, Zhengzhou, 450046 China; 2 International Joint Research Laboratory for Zoonotic Diseases of Henan, Zhengzhou, 450046 China

**Keywords:** Intestinal protozoa parasites, Intestinal microbes, Metagenomic, Beef calves, Fungi, Diarrhea

## Abstract

**Background:**

A close connection between a protozoan parasite and the balance of the other gut microbes of the host has been demonstrated. The calves may be naturally co-infected with many parasites, and the co-effects of parasites on other intestinal microbes of calves remain unclear. This study aims to preliminarily reveal the relationship between intestinal parasites and other intestinal microbes in calves.

**Methods:**

Fecal samples were collected from four calves with bloody diarrhea, four calves with watery diarrhea, and seven normal calves, and the microbial flora of the samples were analyzed by whole-genome sequencing. Protozoal parasites were detected in the metagenome sequences and identified using polymerase chain reaction (PCR).

**Results:**

*Cryptosporidium*, *Eimeria*, *Giardia*, *Blastocystis*, and *Entamoeba* were detected by metagenomic analysis, and the identified species were *Giardia duodenalis* assemblage E, *Cryptosporidium bovis*, *Cryptosporidium ryanae*, *Eimeria bovis*, *Eimeria subspherica*, *Entamoeba bovis*, and *Blastocystis* ST2 and ST10. Metagenomic analysis showed that the intestinal microbes of calves with diarrhea were disordered, especially in calves with bloody diarrhea. Furthermore, different parasites show distinct relationships with the intestinal microecology. *Cryptosporidium*, *Eimeria*, and *Giardia* were negatively correlated with various intestinal bacteria but positively correlated with some fungi. However, *Blastocystis* and *Entamoeba* were positively associated with other gut microbes. Twenty-seven biomarkers not only were significantly enriched in bloody diarrhea, watery diarrhea, and normal calves but were also associated with *Eimeria*, *Cryptosporidium*, and *Giardia*. Only *Eimeria* showed a distinct relationship with seven genera of bacteria, which were significantly enriched in the healthy calves. All 18 genera of fungi were positively correlated with *Cryptosporidium*, *Eimeria*, and *Giardia*, which were also significantly enriched in calves with bloody diarrhea. Functional genes related to parasites and diseases were found mainly in fungi.

**Conclusions:**

This study revealed the relationship between intestinal protozoan parasites and the other calf gut microbiome. Different intestinal protozoan parasites have diametrically opposite effects on other gut microecology, which not only affects bacteria in the gut, but also is significantly related to fungi and archaea.

**Graphical Abstract:**

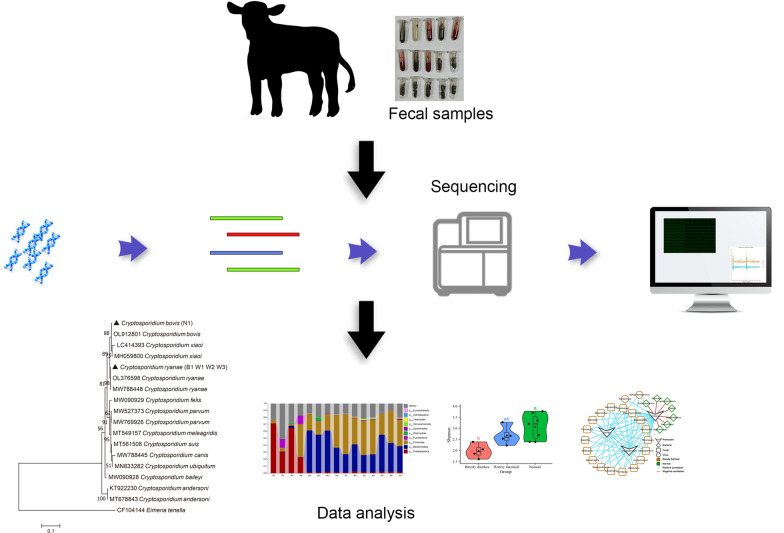

**Supplementary Information:**

The online version contains supplementary material available at 10.1186/s13071-023-05877-z.

## Background

Protozoan parasites are commonly found in the digestive tract of cattle, especially in the rumen and intestines, and have an important effect on their health. Ciliate species, mainly in the rumen, play an important role in rumen fermentation and the stability of rumen ecology [1]. On the contrary, protozoan parasites in the cattle gut are usually associated with intestinal diseases. Many intestinal protozoan parasites, such as *Cryptosporidium*, *Eimeria*, and *Giardia*, cause intestinal disease outbreaks in humans and animals [2, 3]. However, the controversial pathogenicity of some intestinal protozoan parasites, such as *Blastocystis* and nonpathogenic *Entamoeba*, is mainly observed in asymptomatic individuals [4]. Protozoan parasites interact with other gut microbes in symbiotic environments and co-evolve over time [5, 6]. At present, studies on the effects of parasites on calves mainly focus on the interaction between parasites and hosts, and there are few studies on the effects of parasites on the intestinal microflora of calves.

Intestinal microecology impacts cattle health and is correlated with nutrient metabolism, energy production, and the immune system [7]. Intestinal diseases are among the most important health problems in calves (0–1 year of age). Approximately 4–25% of calves in the United States die from diarrhea each year, causing tremendous economic losses to the cattle industry [8–10]. Although many factors cause calf diarrhea, disorders of the intestinal microbiome are the main manifestations of calf diarrhea [10–12]. Diverse gut microbiota prevent colonization by foreign pathogens and enhance the host immune system through interactions between antigens and immune cells during the early stages of life [8]. Studies have shown that restoring the intestinal microbial composition of diarrheal calves by fecal microbiota transplantation can ameliorate diarrhea in pre-weaning calves [13].

There are few effective drugs and vaccines for protozoan parasites such as *Cryptosporidium* and *Giardia duodenalis* [3, 14]. Changes in the intestinal bacteria may be related to various intestinal protozoan parasites, and understanding the effects of parasites on gut microbes could provide new insights into treating parasitic diseases [15–17]. In this study, parasites and other intestinal microecological elements in the posterior intestines of calves were characterized using metagenomic analysis. The association between intestinal protozoan parasites, other intestinal microbes, and calf diarrhea was also investigated, which provides a theoretical basis for maintaining calf intestinal health.

## Methods

### Animal management

Samples for this study were collected from a beef cattle farm in Henan Province, China. A total of 15 fresh stool samples were collected from four calves with bloody diarrhea (B1–B4), four calves with watery diarrhea (W1–W4), and seven calves with normal stools (N1–N7). All calves were 2–3 months old, and samples were collected by rectal sampling, stored in 2-ml cryotubes, transported on dry ice to the laboratory, and stored at −80 °C until use.

### Sample preparation and Illumina sequencing

Total DNA from the fecal microbiota was extracted using the QIAamp Fast DNA Stool Mini Kit (QIAGEN, Hilden, Germany) according to the manufacturer’s instructions (QIAamp Fast DNA Stool Mini Kit Handbook, www.qiagen.com/handbooks). The degree of degradation and potential contamination of DNA was analyzed by electrophoresis using 1% agarose gel. DNA purity was determined using a NanoPhotometer^®^ spectrophotometer (IMPLEN, Westlake Village, CA, USA), and DNA concentration was measured using the Qubit^®^ double-stranded DNA (dsDNA) Assay Kit on a Qubit^®^ 2.0 Fluorometer (Life Technologies, Carlsbad, CA, USA). One microgram of the qualified DNA was used to construct the library. DNA samples were fragmented to 350 base pairs (bp) by sonication, and the DNA fragments were end-polished, A-tailed, and ligated with a full-length adaptor for Illumina sequencing with further polymerase chain reaction (PCR) amplification. The libraries were analyzed for size distribution using an Agilent 2100 Bioanalyzer (Agilent Technologies, Santa Clara, CA, USA) and quantified via real-time PCR. Libraries were sequenced using the Illumina PE150 platform (Illumina, Inc., San Diego, CA, USA).

### Identification of intestinal microorganisms

Metagenomic analysis was carried out following previously published methods [18, 19]. The host sequence was removed from the raw data using Bowtie 2 (v2.4.5) [20] and assembled using MEGAHIT (v1.2.9) [21]. The sequences (≥ 500 bp) were used to predict the open reading frame using MetaGeneMark (v3.38) [22] and eliminate redundancy using CD-HIT (v4.5.8) [23]. Clean data from each sample were mapped to the initial gene catalog using Bowtie2. The corresponding relative abundance of each gene was calculated based on the following formula: *Ai* = *Ci*/*∑*^*n*^_*i=1*_ *Ci* (where *Ni* represents the number of reads mapped to each gene and *Li* represents the length of each gene; *Ci = Ni/Li*) [24]. The obtained genes were used to BLAST the sequences for bacteria, fungi, archaea, viruses, and intestinal protozoan parasites, which were extracted from the National Center for Biotechnology Information (NCBI) non-redundant (NR) database (https://www.ncbi.nlm.nih.gov) using DIAMOND software (v2.0.14) [25]. We used the lowest common ancestor (LCA) algorithm to obtain the number of genes and abundance information for each sample in each taxonomic hierarchy (kingdom, phylum, class, order, family, genus, and species) [26]. DIAMOND software was used to annotate the unigenes using the Kyoto Encyclopedia of Genes and Genomes (KEGG) database (http://www.kegg.jp/kegg/).

### PCR amplification and sequence analysis

Further specialization of parasites was accomplished using PCR. *Giardia* was identified based on the β-giardin (*bg*) gene [27], and *Cryptosporidium* [28], *Eimeria* [29], *Blastocystis* [30]*,* and *Entamoeba* [31] were identified based on the small subunit (*SSU*) ribosomal RNA (rRNA) gene. After amplification, the DNA fragments were separated by agarose gel electrophoresis (1% agarose), stained with DNA Green (TIANDZ, Beijing, China), and observed using a Tanon 3500 gel imaging system (TANON, Shanghai, China). Amplified samples with the target band were selected as positive PCR production. Positive PCR amplicons with the target band were sequenced by SinoGenoMax (Beijing, China). Bidirectional sequencing was chosen to ensure the veracity of sequences. Phylogenetic analysis was conducted using MEGA 7.0 software (http://www.megasoftware.net/), choosing the maximum composite likelihood model, and bootstrap values were calculated by analyzing 1000 replicates.

### Statistical analysis and visualization

Venn diagrams, alpha diversity (Chao1 and Shannon indices), and principal coordinates analysis (PCoA) based on the Bray–Curtis distance were calculated and plotted using Tutools (https://www.cloudtutu.com), and the ImageGP (https://416h86i955.zicp.fun/Cloud_Platform/front/#/) platform, and microbial features showing differential abundance were identified using linear discriminant analysis effect size (LEfSe) threshold criteria of linear discriminant analysis (LDA) score > 2 and *P* < 0.05 (http://huttenhower.sph.harvard.edu/galaxy/). To reveal the relationship between parasites and the microbial or KEGG ortholog group (KO), we calculated the pairwise Spearman’s rank correlation and removed coefficients below 0.7 with *P* > 0.05. We adjusted the *P*-value to avoid false positives using the Benjamin-Hochberg (BH) method from the ‘Hmisc’ package in R (v4.2.2) statistical software. Network analysis and visualization were conducted using Gephi (https://gephi.org/) and Cytoscape (https://cytoscape.org/).

## Results

### Species of intestinal protozoan parasites

**Fig. 1:**
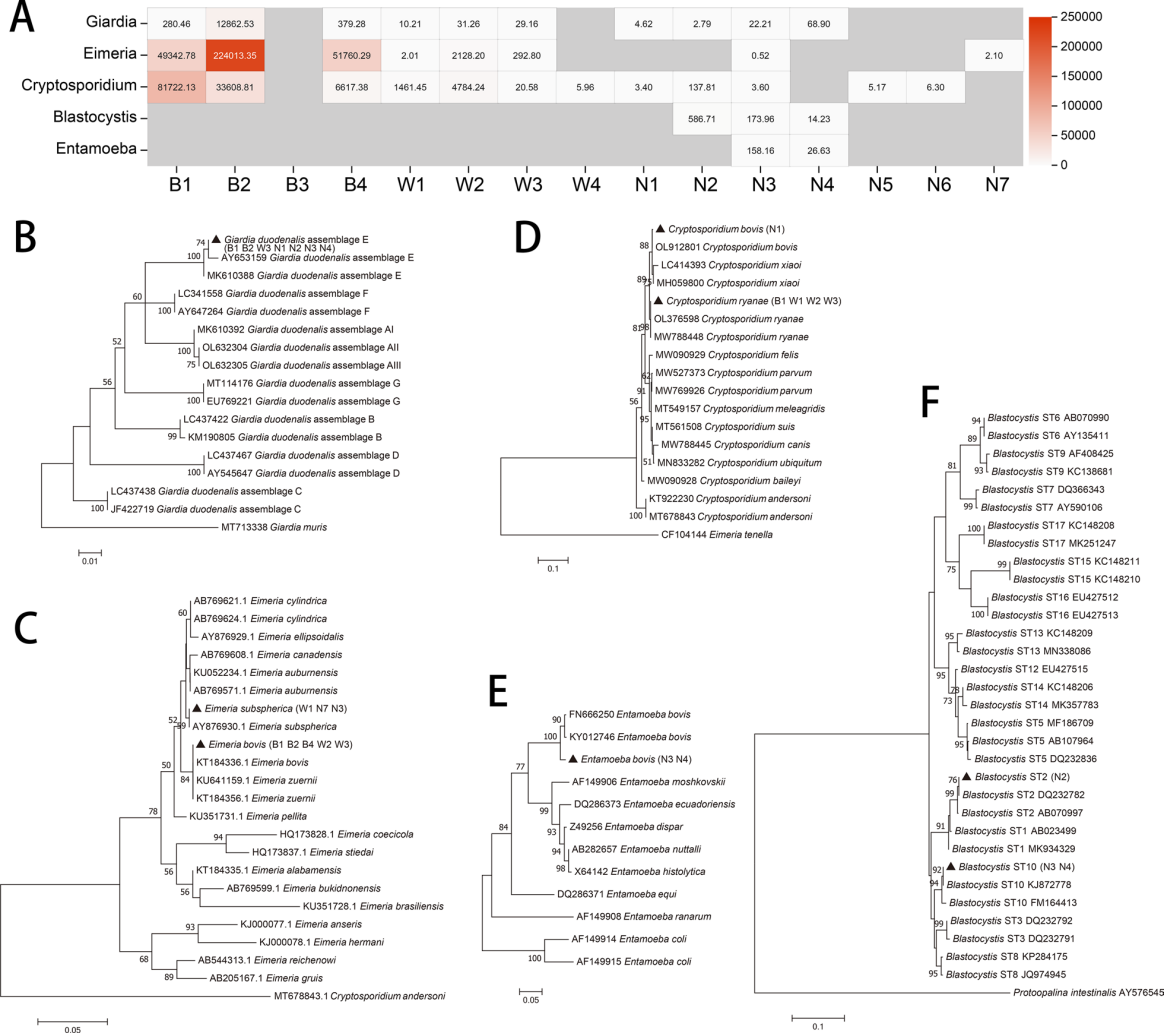
Intestinal parasitic protozoa detected in the calves. **a** Parasites annotated by metagenomic sequencing. **b** Phylogenetic relationships of *Giardia duodenalis* based on β-giardin (*bg*) nucleotide sequences. **c** Phylogenetic relationships of *Eimeria* based on the *SSU* rRNA gene. **d** Phylogenetic relationships of *Cryptosporidium* based on the *SSU* rRNA gene. **e** Phylogenetic relationships of *Blastocystis* based on the *SSU* rRNA gene. **f** Phylogenetic relationships of *Entamoeba* based on the *SSU* rRNA gene. Phylogenetic relationships were calculated using the maximum composite likelihood model. Percent bootstrap values greater than 50% from 1000 replicates are shown next to the branches. Triangles represent isolates detected in this study, and the name of samples which detected the parasite are written in parentheses

The parasites identified using metagenomics belonged to the genera *Giardia*, *Eimeria*, *Cryptosporidium*, *Blastocystis*, and *Entamoeba* (Fig. 1A). Specific genetic information is shown in Additional file 3: Table S2. Not all positive samples were successfully amplified. Based on these results, the parasite species in calves were *G. duodenalis* assemblage E (Fig. 1B), *Cryptosporidium bovis*, *Cryptosporidium ryanae* (Fig. 1C), *Eimeria bovis*, *Eimeria subspherica* (Fig. 1D), *Blastocystis* (ST2 and ST10) (Fig. 1E), and *Entamoeba bovis* (Fig. 1F).

### Intestinal microbial imbalance in diarrheal calves

**Fig. 2:**
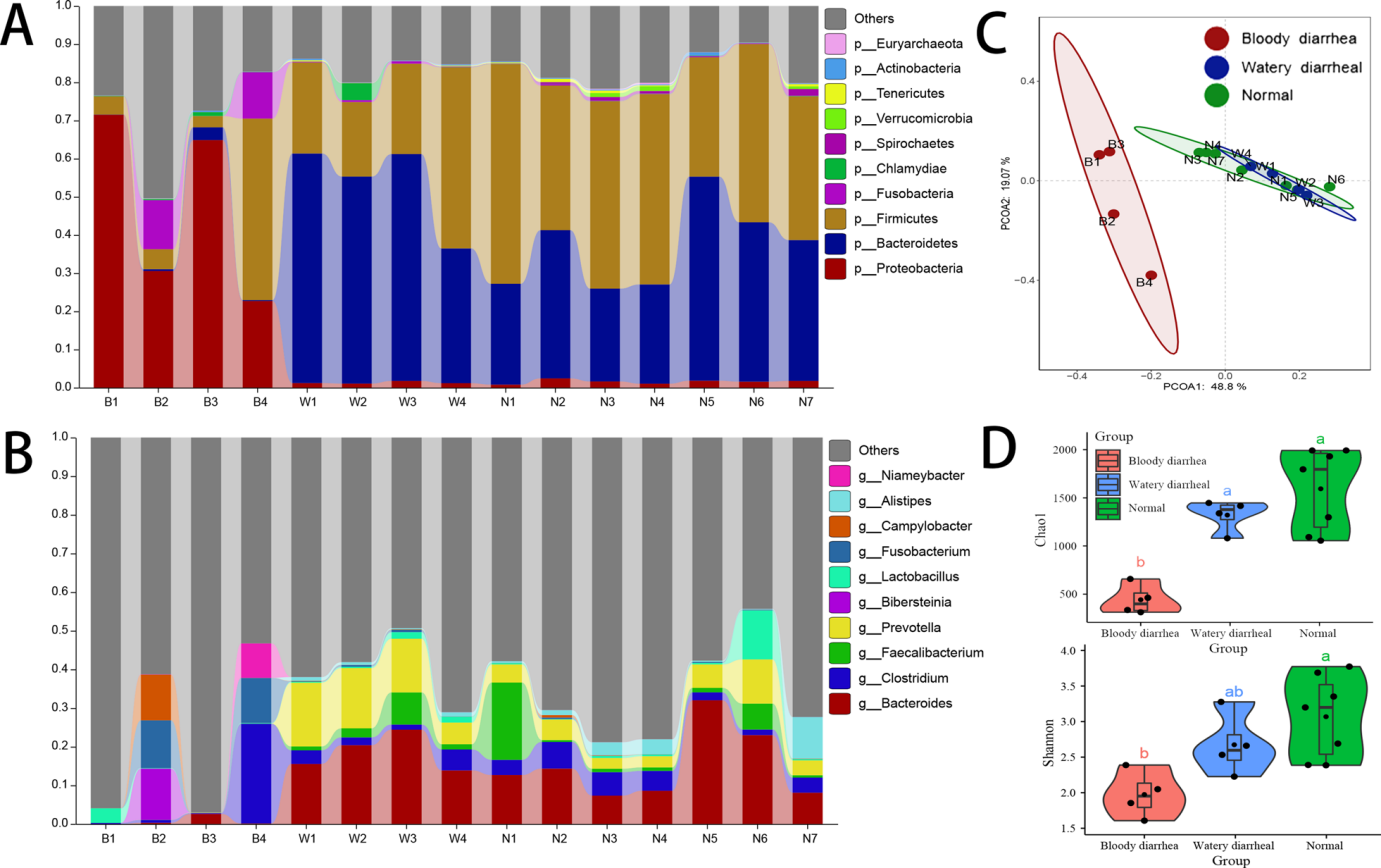
Comparison of gut microbes among calves with bloody diarrhea, watery diarrhea, and normal stools. **a** Relative abundance (%) at the phylum level. **b** Relative abundance (%) at the genus level. **c** PCoA of gut microbes based on the Bray–Curtis distance. **d** α-diversity (Chao1 and Shannon index) of gut microbes. Boxes of α-diversity denote the interquartile range (IQR) between the first and third quartiles (the 25th and 75th percentiles, respectively), and the line inside denotes the median. Whiskers denote the lowest and highest values within 1.5 times and the IQR from the first and third quartiles, respectively

Comparing calves with watery diarrhea and calves with normal stools, the major intestinal microbiota of calves with bloody diarrhea changed greatly at the phylum and genus levels. At the phylum level, *Firmicutes*, *Bacteroidetes*, and *Proteobacteria* were the main phyla in the gut of the calves. *Firmicutes* and *Bacteroidetes* were mainly found in calves with watery diarrhea and calves with normal stools, but *Proteobacteria* were more abundant in calves with bloody diarrhea (Fig. 2A). At the genus level, *Bacteroides* were mainly found in calves with watery diarrhea and calves with normal stools and were lower in calves with bloody diarrhea (Fig. 2B).

The intestinal microbes of calves with diarrhea show disorders, especially calves with bloody diarrhea. The PCoA results show that calves with watery diarrhea and calves with normal stools have comparable intestinal microbiota compositions, which differ significantly from calves with bloody diarrhea (Fig. 2C). The diversity of microbes declined with an increase in the degree of diarrhea, while in calves with bloody diarrhea, it was significantly reduced (Fig. 2D).

The taxa that most likely explain the differences between calves with bloody diarrhea, calves with watery diarrhea, and calves with normal stools were defined by LEfSe. One hundred thirty-six biomarkers were detected at the genus level (LDA > 2, *P* < *0.05*). Thirty-two biomarkers significantly enriched in calves with bloody diarrhea, including various fungi and opportunistic pathogens, such as *Escherichia*, *Streptococcus*, *Salmonella*, and *Shigella*. Thirty-two biomarkers in calves with watery diarrhea, mainly *Bacteroidetes*, *Firmicutes*, and *Proteobacteria*, and 71 biomarkers were significantly enriched in calves with normal stool and mainly belonged to *Firmicutes* (Additional file 1: Fig. S1).

### Relationship between parasites, other microbes, and calf diarrhea

*Cryptosporidium*, *Eimeria*, and *Giardia* were distributed in both diarrheal and healthy calves and were more abundant in diarrheal cattle and lower in calves with normal stools. However, *Blastocystis* and *Entamoeba* were found only in calves with normal stools, with a higher abundance than that of the other parasites (Additional file 1: Fig. S2).

**Fig. 3:**
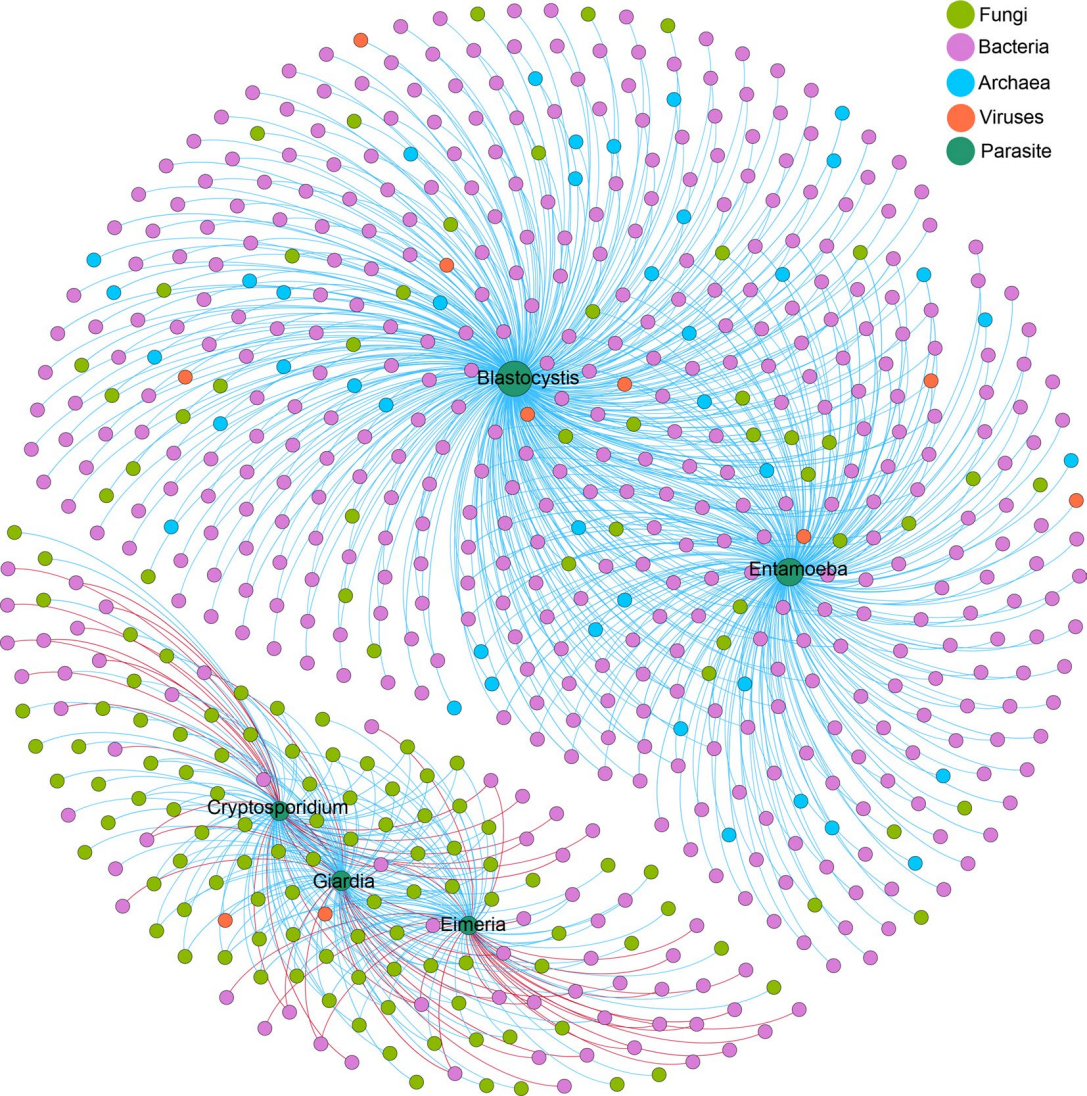
Relationship between protozoan parasites and calf microbiome. Network relationships between protozoan parasites and gut microbes at the genus level. The relationship was calculated using the pairwise Spearman rank correlation, the removed coefficient was below 0.7, *P* > 0.05, and the *P*-value was adjusted to avoid false positives using the BH method

The network analysis results showed that the parasites were associated with various other gut microbes (Fig. 3). In total, five protozoan parasites formed two distinct networks, indicating that protozoan parasites have different mutual regulatory relationships with other intestinal microbes. *Blastocystis* and *Entamoeba* were related to the diversity of the intestinal bacteria, archaea, fungi, and viruses in calves. All 494 genera of intestinal microbes were positively associated with *Blastocystis* and *Entamoeba*; about 81.2% (401/494) were bacteria, and the others were archaea (41/494), fungi (44/494), and viruses (8/494). Interestingly, *Blastocystis* and *Entamoeba* were significantly positively correlated with various archaea, including many methanogens: *Methanothermus*, *Methanothermococcus*, *Methanothermobacter*, *Methanosalsum*, and *Methanolacinia*. One hundred sixty-one intestinal microbes were associated with *Eimeria*, *Cryptosporidium*, and *Giardia*, including 62 genera of bacteria, 97 fungi, and two viruses. Most of the bacteria were negatively correlated with *Eimeria*, *Cryptosporidium,* and *Giardia*, and all the fungi and viruses were positively correlated with them, meaning that these protozoan parasites may be related to the imbalance of other gut microbes.

### Key microbes associated with protozoan parasites and the health of calves

**Fig. 4:**
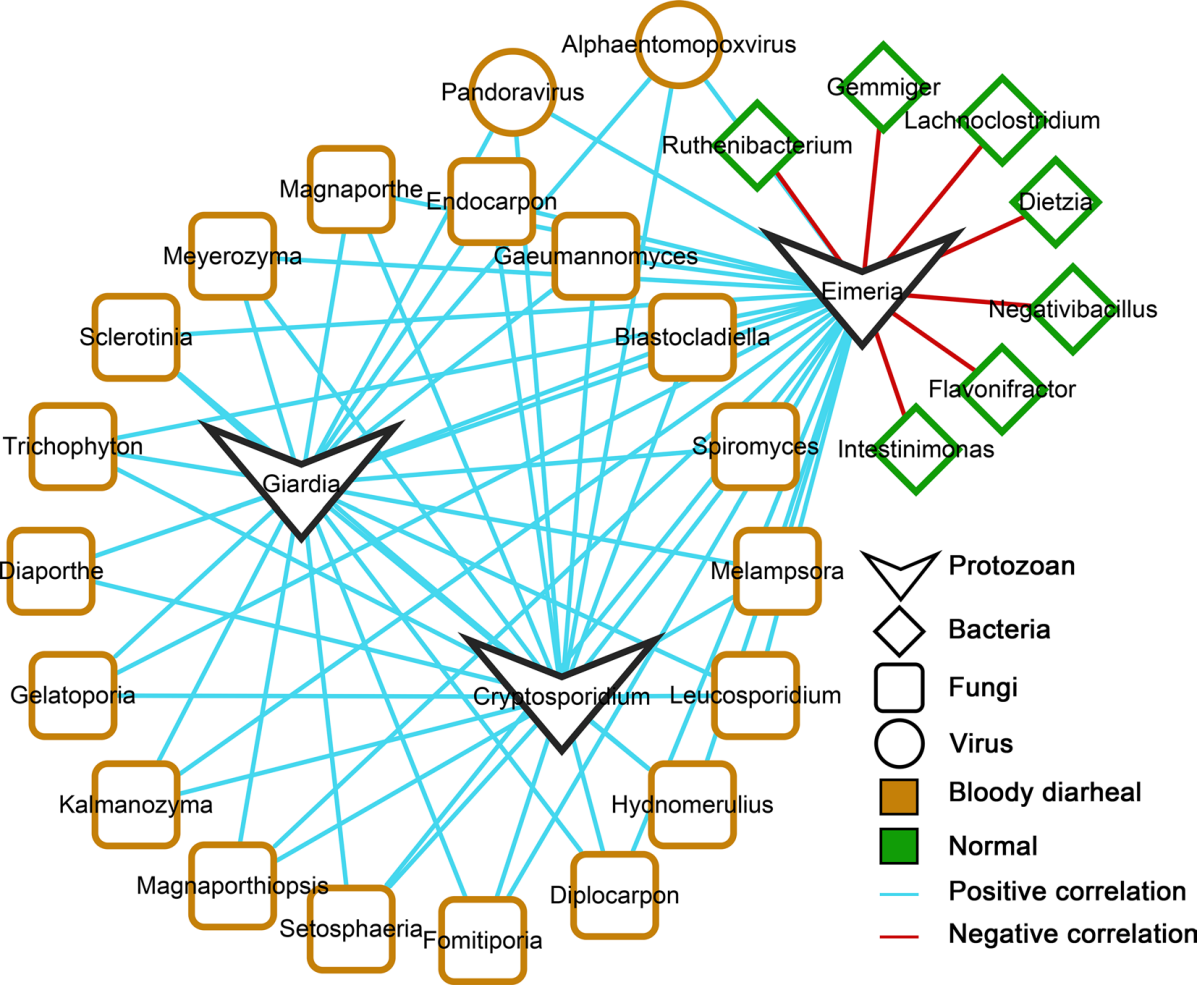
Microbes that not only are associated with parasites but also vary significantly in different degrees of diarrhea

Twenty-seven biomarkers were not only significantly enriched in calves with bloody diarrhea, watery diarrhea, and normal stools, but were also associated with *Eimeria*, *Cryptosporidium*, and *Giardia*. In addition, all intestinal microorganisms associated with *Blastocystis* and *Entamoeba* were not significantly enriched in calves with varying degrees of diarrhea. Among the biomarkers, seven genera of bacteria were negatively correlated with *Eimeria*, which were mainly enriched in the intestines of calves with normal stools, namely, *Dietzia*, *Flavonifractor*, *Gemmiger*, *Intestinimonas*, *Lachnoclostridium*, *Negativibacillus*, and *Ruthenibacterium.* All 18 genera of fungi were positively correlated with *Eimeria*, *Cryptosporidium,* and *Giardia* were enriched in the intestines of calves with bloody diarrhea, including *Blastocladiella*, *Diaporthe*, *Diplocarpon*, *Endocarpon*, *Fomitiporia*, *Gaeumannomyces*, *Gelatoporia*, *Hydnomerulius*, *Kalmanozyma*, *Leucosporidium*, *Magnaporthe*, *Magnaporthiopsis*, *Melampsora*, *Meyerozyma*, *Sclerotinia*, *Setosphaeria*, *Spiromyces*, and *Trichophyton*. The two viruses, *Alphaentomopoxvirus* and *Pandoravirus*, were enriched in the intestines of calves with bloody diarrhea (Fig. 4).

### Relationship between parasites and other gut microbial function

Functional annotation based on the KEGG database showed that the intestinal microorganisms of calves had abundant functional genes (Additional file 1: Fig. S3). The functional categories identified included Metabolism, Cellular Processes, Environmental Information Processing, Genetic Information Processing, Human Diseases, and Organismal Systems. PCoA results showed that, at the KO level, the samples of calves with watery diarrhea and normal stools showed a clear distance from calves with bloody diarrhea. This suggests that severe diarrhea could affect gut microbial function (Additional file 1: Fig. S4).

**Fig. 5:**
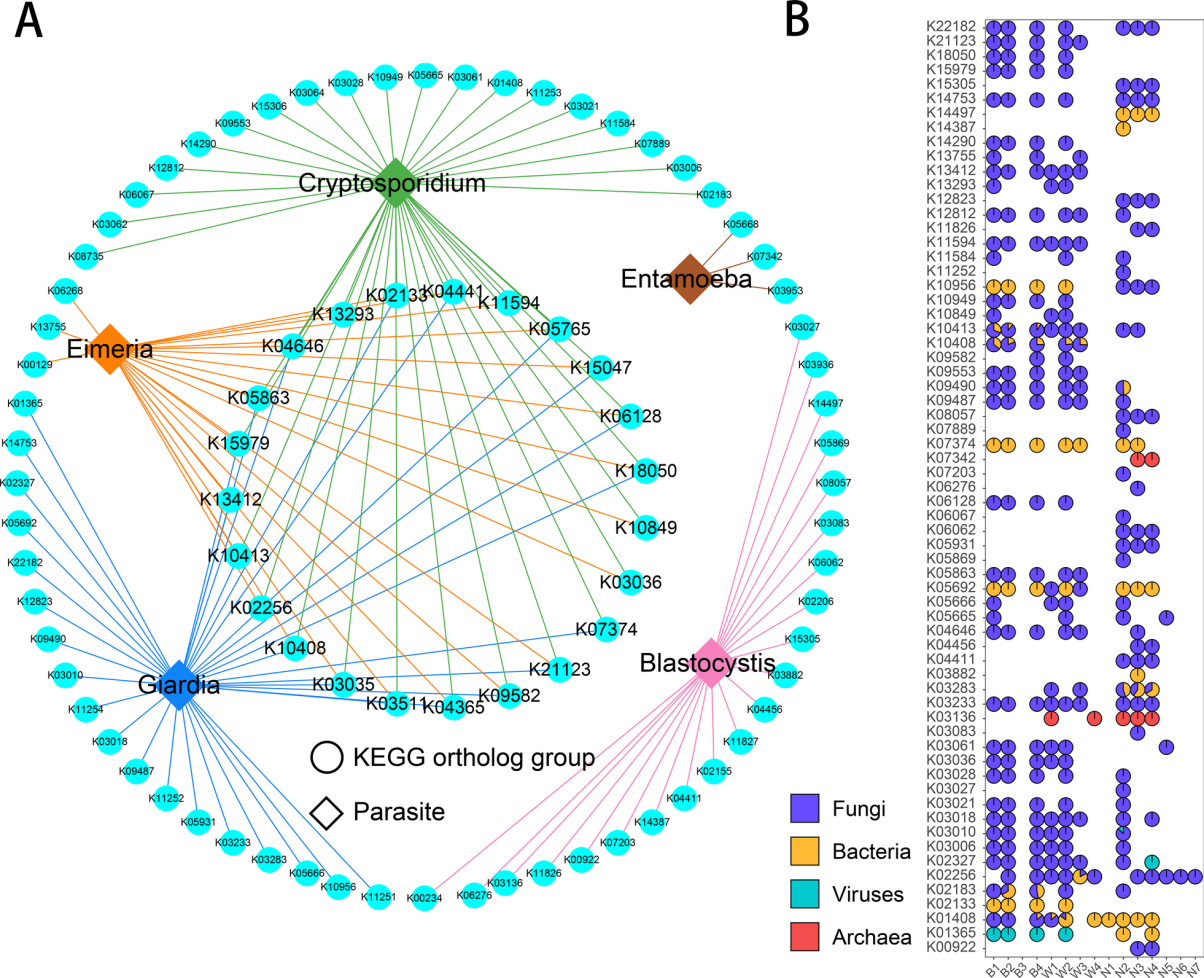
Key pathway associated with protozoan parasites and calf diarrhea. **a** Disease- and parasite-related KOs. **b** Carriers of KOs associated with diseases and parasites

The functions of other gut microbes were related to intestinal parasites. A total of 611 KOs were found to be associated with parasites by calculating their correlation coefficients (Additional file 2: Table S1), and 87 KOs were disease-related (Fig. 5A). The genes annotated as 87 KOs were aligned with the NCBI NR database to trace the possible integration of the bacteria. Sixty-six KOs were successfully annotated, most of which were from fungi (Fig. 5B).

## Discussion

Intestinal protozoan parasitic diseases are prevalent in ruminants, and infections with intestinal protozoan parasites in cattle are associated with outbreaks of diarrhea, mainly in calves, leading to economic losses for agricultural producers [32, 33]. However, the relationship between parasites and calf gut microbes remains unclear. Here, we have shown the relationship between parasites, gut microbes, and diarrhea.

The abundance of parasites found in this study was associated with calf diarrhea. *Eimeria*, *Cryptosporidium*, and *Giardia* were detected in both diarrheal and normal calves, but in higher abundance in diarrheal calves. Previous studies have shown similar results; the oocysts/cyst per gram of feces of *Eimeria*, *Cryptosporidium*, and *Giardia* in diarrheal animals were positively higher than in healthy animals [34–36]. Nevertheless, *Blastocystis* and *Entamoeba* were found only in healthy calves, which may indicate a positive association with intestinal health. The pathogenicity of *Blastocystis* and *Entamoeba* in cattle is controversial; however, *Blastocystis* and *Entamoeba* are highly prevalent in cattle and are mostly detected in healthy cattle [37, 38], which is consistent with the results of this study.

Calf diarrhea was often characterized by an imbalance in intestinal flora [10–12], and this study also revealed intestinal microbial imbalance in calves with diarrhea. Moreover, *Eimeria*, *Cryptosporidium*, and *Giardia intestinalis* were related to an imbalance in gut microbes. Previous studies have also shown negative effects of these parasites on gut microbes. For example, *Eimeria* damaged the chicken intestinal barrier and reduced the abundance of probiotic bacteria [16]. *Cryptosporidium* infection decreased bacterial diversity [39], and *Giardia* infection was associated with significant dysbiosis within the murine foregut and hindgut [15]. By contrast, *Blastocystis* and *Entamoeba* in this study were associated with the diversity of other intestinal microorganisms. The same phenomenon has been observed in humans, where it was found that *Blastocystis* was associated with both higher richness and higher evenness of the gut bacterial microbiota, whereas *Entamoeba* was associated only with higher richness, all of which have a role in maintaining intestinal health [17]. It is worth noting that *Blastocystis* and *Entamoeba* were significantly positively correlated with various methanogenic archaea. The possible use of methanogens as probiotics has received particular attention in humans [40, 41]; therefore, the positive relationship between *Blastocystis*, *Entamoeba*, and methanogenic archaea may be beneficial for gut health. The relationship between archaea and parasites in animals has received little attention, and more research is needed to uncover this phenomenon.

Little attention has been paid to the relationship between fungi and parasites in cattle. In this study, many fungi that were significantly enriched in calves with bloody diarrhea were positively correlated with *Eimeria*, *Cryptosporidium*, and *Giardia*. The parasites and fungi may play a synergistic role in intestinal disease in calves. Previous studies have found that higher fungal abundance is associated with intestinal disease, with a higher prevalence and abundance in patients with inflammatory bowel disease [42], which enhances inflammation by preventing intestinal healing [43, 44]. Fungi are neglected microorganisms that influence intestinal diseases in cattle, and further studies on the interactions between fungi and parasites are needed.

## Conclusion

In conclusion, this study revealed the relationship between protozoan parasites and the calf microbiome. *Eimeria*, *Cryptosporidium*, and *Giardia* are associated with calf diarrhea and intestinal microbial disorders. By contrast, *Blastocystis* and *Entamoeba* play positive roles in maintaining intestinal health and microbial diversity. In addition, many fungi have a potential synergistic relationship with *Eimeria*, *Cryptosporidium*, and *Giardia*; on the contrary, archaea were only positively correlated with *Blastocystis* and *Entamoeba*. Due to the limited collection from the same farm, more extensive sampling in the future will clearly help produce a better association between protozoan parasites and intestinal health. As we described only the association of protozoan parasites, microbiome, and health of calves in this study, further work, including intervention studies, will be needed to fully elucidate the role of the protozoan parasites in the intestinal environment.

## Supplementary Information

**Additional file 1: Figure S1.** LEfSe analysis of gut microbes in calves with bloody diarrhea, watery diarrhea, and normal stools. Microbial features showing differential abundance were identified using the LEfSe threshold criteria of an LDA score > 2 and *P* < 0.05. The length of the bar column represents the LDA score. **Figure S2.** Abundance of protozoan parasites in calves with bloody diarrhea, watery diarrhea, and normal stools. **Figure S3.** KEGG pathway annotation. **Figure S4.** PCoA of gut microbial functions based on Bray–Curtis distance.

**Additional file 2: Table S1.** Correlation coefficients between KEGG ortholog group and parasites.

**Additional file 3: Table S2.** Annotated parasite genes and corresponding species information.

## Data Availability

The data generated during this study are available at the Sequence Read Archive under the BioProject accession number PRJNA924548.

## Abbreviations

KEGG: Kyoto Encyclopedia of Genes and Genomes
KO: KEGG ortholog group
LDA: Linear discriminant analysis
LEfSe: Linear discriminant analysis effect size
PCoA: Principal coordinates analysis
PCR: Polymerase chain reaction
NCBI: National Center for Biotechnology Information
