# Supplementary material for: Metagenomic analysis reveals the relationship between intestinal protozoan parasites and the intestinal microecological balance in calves
Source: Parasit Vectors. 2023 Jul 31;16:257. doi: 10.1186/s13071-023-05877-z (PMC10388496; doi:10.1186/s13071-023-05877-z)

Fig.S1 LEfSe analysis of gut microbes in calves with bloody diarrhea, watery diarrhea, and normal stools. Microbial features showing differential abundance were identified using the LEfSe threshold criteria of an LDA score > 2 and P-value < 0.05. The length of the bar column represents the LDA score.

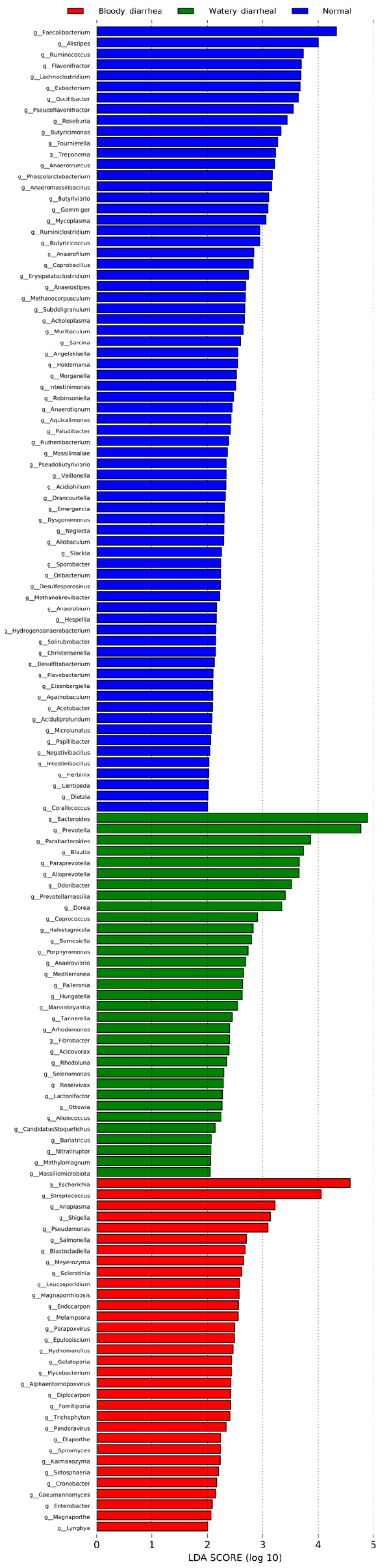

Fig.S2 Absolute abundance of protozoan parasites in calves with bloody diarrhea, watery diarrhea, and normal stools.

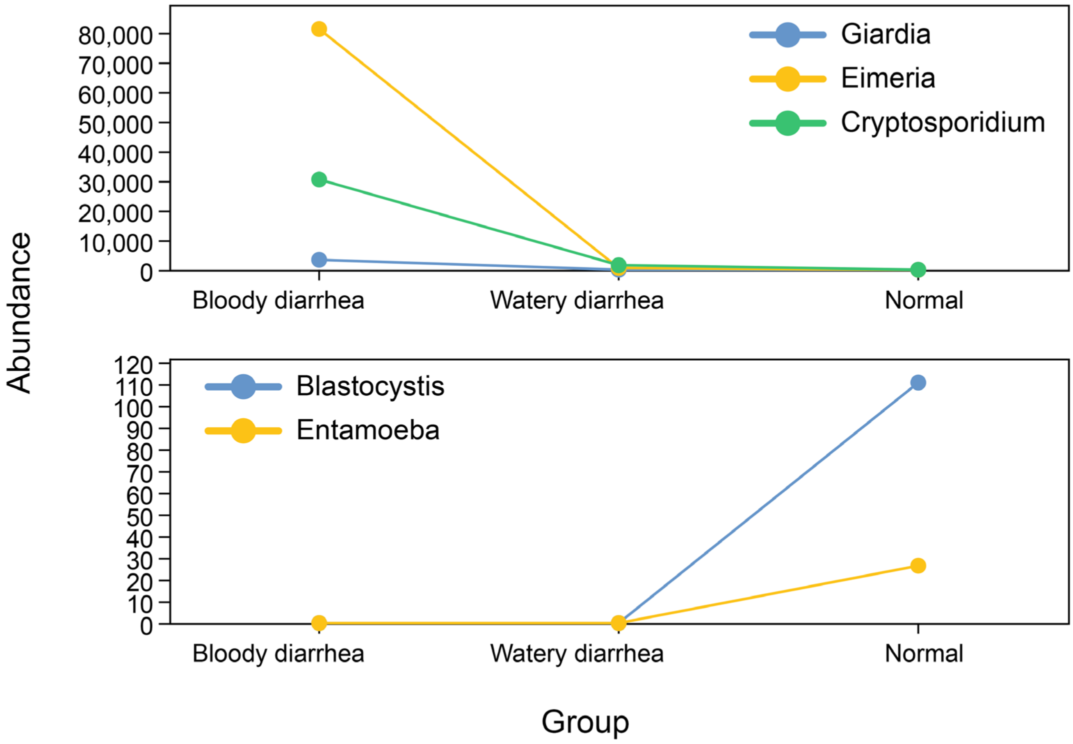

Fig.S3 KEGG pathway annotation.

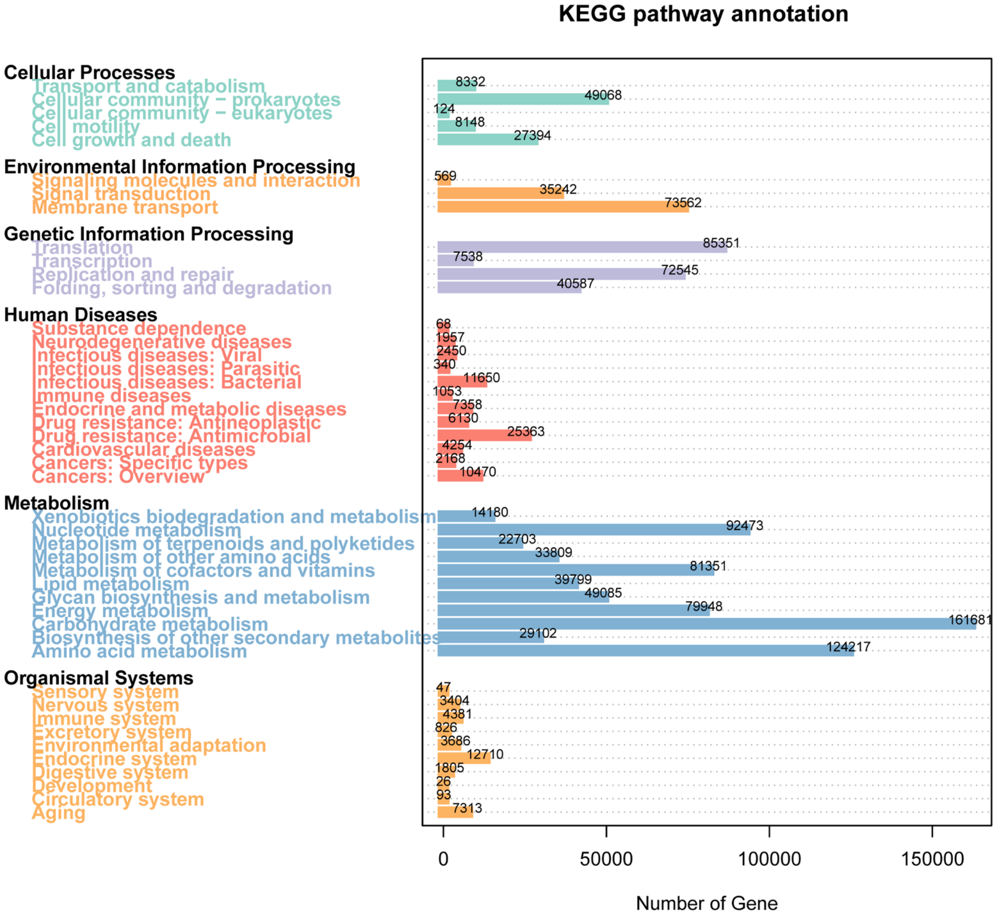

Fig.S4 PCoA of gut microbial functions based on Bray–Curtis distance.

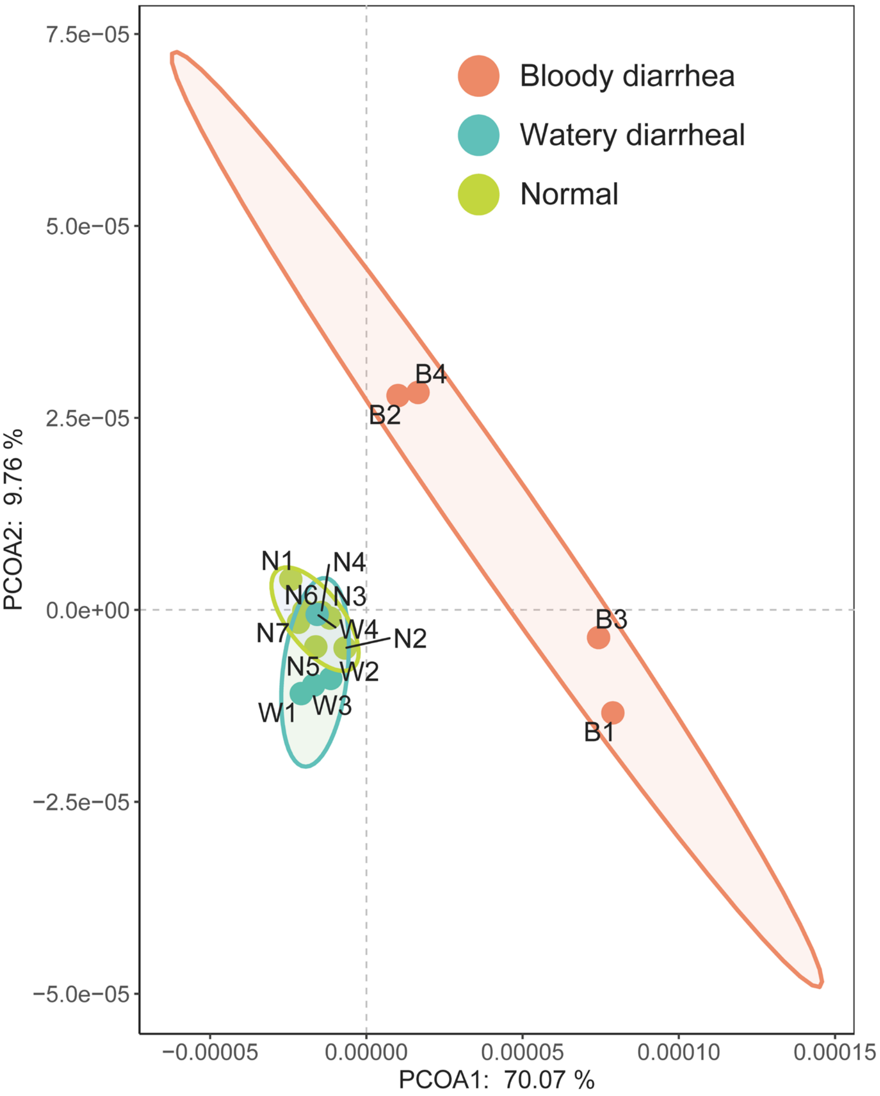

Supplement: Supplementary file 1 — Additional file 1: Figure S1. LEfSe analysis of gut microbes in calves with bloody diarrhea, watery diarrhea, and normal stools. Microbial features showing differential abundance were identified using the LEfSe threshold criteria of an LDA score > 2 and P < 0.05. The length of the bar column represents the LDA score. Figure S2. Abundance of protozoan parasites in calves with bloody diarrhea, watery diarrhea, and normal stools. Figure S3. KEGG pathway annotation. Figure S4. PCoA of gut microbial functions based on Bray–Curtis distance. [file 13071_2023_5877_MOESM1_ESM.pdf]
